# Chemical Distribution Kinetics of Five Polycyclic Aromatic Hydrocarbons in a Microphysiological System

**DOI:** 10.3390/toxics14090837

**Published:** 2026-09-21

**Authors:** Katharina S. Nitsche, Susana Proença, Wouter Bakker, Paul L. Carmichael, Hans Bouwmeester, Nynke I. Kramer

**Affiliations:** 1 Division of Toxicology, Wageningen University & Research, P.O. Box 8000, 6700 EA Wageningen, The Netherlandshans.bouwmeester@wur.nl (H.B.); 2 esqLABS GmbH, 26683 Saterland, Germany; 3 Unilever Safety and Environmental & Regulatory Science Group, Colworth Science Park, Sharnbrook, Bedfordshire MK44 1LQ, UK

**Keywords:** partitioning, mass balance modelling, true dose, organ-on-chip, next-generation risk assessment

## Abstract

As next-generation risk assessment accelerates the shift towards non-animal models, it also necessitates the development of tools that quantify the chemical in vitro distribution to derive an effective concentration. Although several predictive models have already been developed, none have thus far specifically focused on microphysiological systems. Unlike static well plates, microphysiological systems use (micro-) fluidic flow, consist of largely hydrophobic plastic surfaces, and often rely on protein-rich hydrogel matrices for cell scaffolding. However, these properties can alter the nominal concentration in microfluidic systems, thereby affecting the freely available chemical fraction that can reach the cells. Our objective was to measure the mass distribution in the Organoplate 2-lane^®^ in the absence of cells to develop an in silico mass balance model using five polycyclic aromatic hydrocarbons under three conditions: bare (no serum), 2% serum media, and bare medium with Matrigel. Our mass distribution analysis showed significant chemical losses in all conditions over 16 h of exposure due to evaporation and plastic sorption, likely accelerated by media movement and the device characteristics. Next, we modelled the in vitro mass balance kinetics of the five polycyclic aromatic hydrocarbons using both raw and loss-corrected measurement data to derive and compare the rate constants and free/bound fractions. As expected, already 2% serum was the main contributor to chemical binding, leaving no free fraction, while the presence of Matrigel delayed the overall distribution kinetics. Based on these results, we emphasise the need to study in vitro distribution, particularly in microphysiological systems, as the nominal concentration of chemicals may not align with the free fraction that reaches the cells.

## 1. Introduction

The shift away from animal testing in chemical safety assessments has accelerated the adoption of in silico, in chemico, and in vitro methods, collectively known as new approach methodologies (NAMs) [1,2]. NAMs align with the principles of next-generation risk assessment (NGRA), which is designed to be exposure-led, hypothesis-driven, and human-relevant, addressing the ethical and scientific demands of 21st-century toxicity testing [3]. Within NGRA frameworks, human cell-based in vitro models play a central role, as they can provide both concentration–response data and ADME (absorption, distribution, metabolism, and excretion) parameters for in silico modelling to estimate human (non-)toxic doses [4,5]. However, a key challenge in translating in vitro findings into an NGRA decision context is that most assays report effects at nominal concentration (C_nominal_), defined as the amount of chemical added per volume of medium [6]. C_nominal_, however, does not accurately reflect the biologically available concentration at the target site, resulting in misleading quantitative in vitro–in vivo extrapolations (QIVIVE) because QIVIVE assumes that the in vitro nominal concentration metric and the in vivo target nominal concentration are equivalent [7,8]. However, differences in binding processes can lead to disproportionate differences in nominal versus free concentration between the in vitro systems and the target in vivo matrix [9,10,11,12].

To facilitate QIVIVE based on free concentrations, NGRA frameworks increasingly incorporate in silico mass balance models that estimate C_free_ from C_nominal_ in in vitro systems by accounting for processes such as non-specific binding (also known as partitioning, C_bound_) across compartments (e.g., plastic, lipids, serum proteins), as well as processes like evaporation, degradation, transport, metabolism, and bioaccumulation [11,13,14,15]. To determine C_bound_ and C_free_, in silico mass balance models include parameters such as physicochemical properties (logP, Henry’s law constant) and system characteristics (e.g., volume, plastic surface, flow, temperature), combined with mathematically and/or experimentally obtained data (i.e., QSARs, flow rates, measured concentration in serum constituents, plastic, cell lipids, etc.) [16]. The model design, including the choice of compartments, equations, and parameterisation, depends on the intended application, the assumptions made, and the experimental setup, as reviewed by Proença and colleagues [9]. More specifically, equilibrium mass balance models assume steady-state conditions, whereas time-dependent mass balance models use differential equations with rate constants to describe a (thermo-)dynamic equilibrium. Hence, each model has a specific applicability domain (e.g., chemical, culture platform, compartments) [17,18,19]. Current mass balance models primarily focus on cell monolayers in plastic well plates, whereas novel culture platforms, such as microfluidic systems (MPS, also known as organ-on-chip), are rarely studied despite their growing market [18,20].

MPS technology refers to small-scale devices designed for (patho)physiologically relevant cell culturing by introducing shear stress with (micro-) fluidic flow in a partially 3D environment [21]. However, these properties influence chemical mass distribution kinetics: (micro-) fluidic flow enhances mass transfer rates, mainly in hydrophobic polymer-based hardware with a high surface-to-medium ratio, which facilitates binding, as do scaffolds using protein-rich hydrogel matrices (ECM); whereas small medium volumes relative to cell numbers impede diffusion [9,20,22,23,24]. Consequently, there is an urgent need to develop mass balance models for MPS platforms to improve their translatability to QIVIVE and toxicity assessments, and to enable cross-system comparisons.

Our study aimed to characterise the bound/free fractions of five polycyclic aromatic hydrocarbons (PAHs) with varying physicochemical properties (lipophilicity (logP 4.1–6.3) and volatility) in the 2-lane Organoplate MPS. The Organoplate was selected for its distinctive design, which includes gravity-driven flow, a large polystyrene surface, the need for an ECM scaffold, and a small medium volume relative to the number of cells. To determine and refine chemical recovery from the various in vitro compartment matrices (medium, serum, plastic, and ECM), we established a liquid–liquid extraction protocol that enabled direct analysis by gas chromatography-mass spectrometry (GC-MS/MS). Next, we measured the mass distribution over 16 h in three conditions without cells: bare (no serum) medium, 2% serum medium, and bare medium with Matrigel in the extracellular matrix (ECM) channel to derive and fit the rate constants. Finally, we used the mass distribution data to model the mass balance in the Organoplate MPS, allowing prediction of bound and free PAH fractions in the system and extrapolation to the 10% medium condition with cells.

### Theory

Our time-dependent mass balance model extends the previous steady-state equilibrium partition model by Kramer [25] developed for PAHs and the time-dependent model by Stadnicka-Michalak and colleagues [12]. Combining both, we (1) created a mass balance model for five PAHs with system parameters tailored to the 2-lane Organoplate MPS, (2) fitted plastic and ECM rate constants with the experimental data from the Organoplate, (3) used the rate constants from (2) to determine the bound and free fractions and (4) extrapolated our model for a theoretical toxicity assay application with HepaRG cells and 10% FCS to compare the free fractions. The model was written and simulated in R (version 2025.09.01), using the RStudio interface (https://github.com/nitsc005/Mass-Distribution, accessed on 13 July 2026).

**Figure 1:**
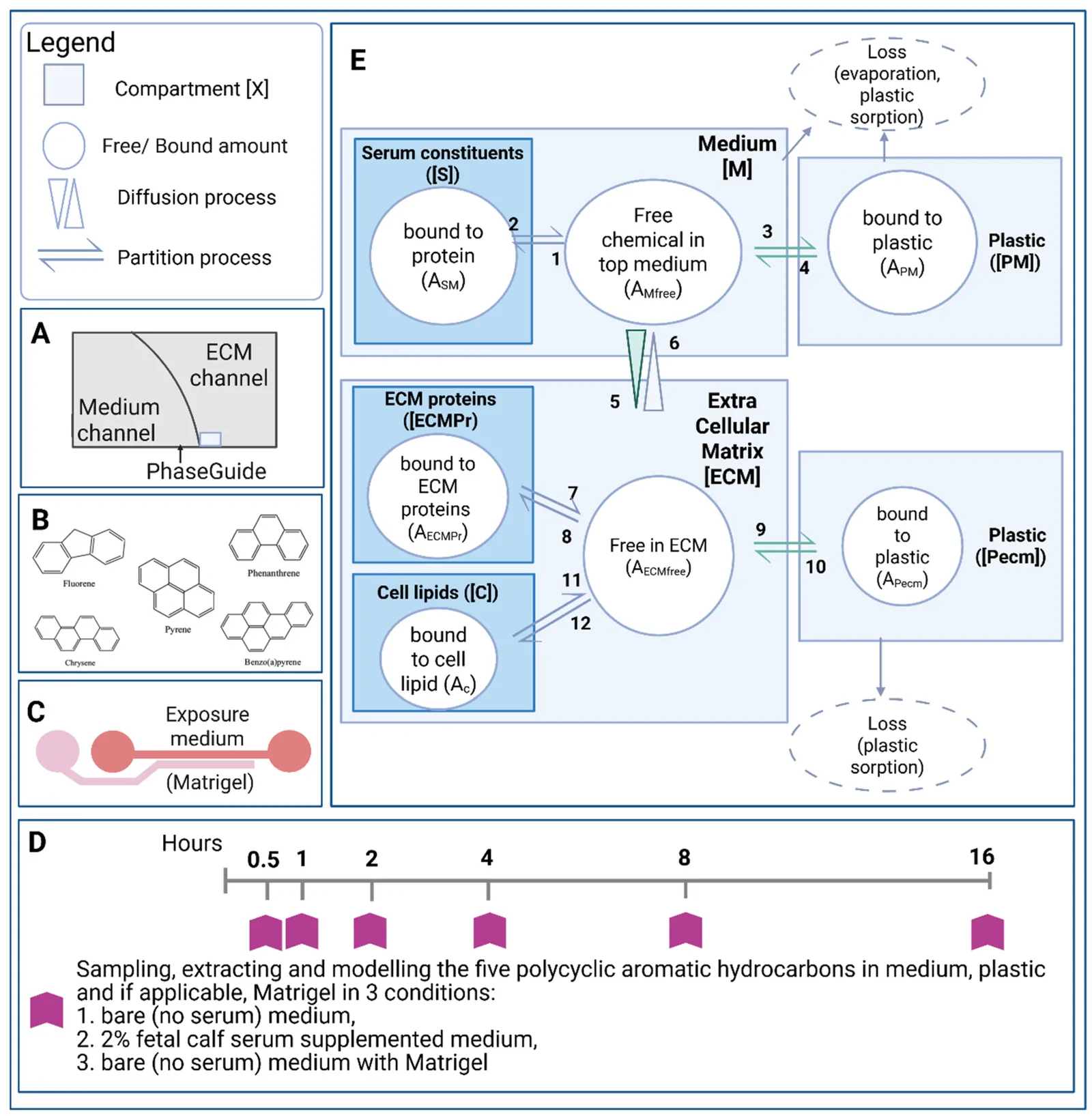
Schematics of the Organoplate 2-lane microphysiological system and study design. (**A**) Cross section of a chip illustrating the channels with a Phaseguide, a short ridge of plastic separating the medium and ECM by acting as a capillary pressure barrier. (**B**) Polycyclic aromatic hydrocarbons used in this study: fluorene, phenanthrene, pyrene, chrysene and benzo[a]pyrene (b[a]p). (**C**) Exposure setup in an individual chip. (**D**) Sampling timeline in the three conditions: bare (no serum) exposure medium, bare exposure medium supplemented with 2% foetal calf serum (FCS) and bare exposure medium with Matrigel in the ECM channel. (**E**) Schematic representation of the mass balance model based on Organoplate. Depicted are the in vitro compartments ([X], medium, serum, plastic, extracellular matrix proteins, plastic, cell lipids), binding/partitioning processes (arrows 1–12) and diffusion (triangles 5 and 6) processes with fitted parameters highlighted in green, as well as the resulting free and bound amounts (circles). Loss (no number) indicates potential losses due to evaporation and plastic sorption.

Based on the device layout (Figure 1) and the system components, we tailored the mass distribution model to the Organoplate MPS that consists of a medium perfusion channel ([M]) and an ECM channel ([ECM]). To simplify the compartments, surrogates were used to represent the biological materials, as these consist of numerous different types of proteins and lipids [26,27]. The compartments (see Figure 1A) were based on the following general surrogates: foetal calf serum (FCS, here: [S],) for all serum constituents [26] in the medium channel, total plastic (here: [PM] + [Pecm] = [P]) for the polar polymer material polystyrene [28] and ECM protein for all hydrogel constituents in Matrigel (here: [ECMPr]) [29]. For the cell-based extrapolation, the polar cell (membrane) lipids were included to represent the phospholipid liposomes [26] (here: [C]).

To model the time-dependent distribution kinetics and fit the rate constants with the experimental data, we first defined the free concentration Equation (1) in the medium channel (C_freeM_, nmol/mm^3^), which depends on the nominal concentration in the medium channel (nominal amount (nmol) divided by the media volume (mm^3^)) and the partitioning (K_Serum_, nmol FCS/mm^3^ medium) of the serum constituents concentration [S] (nmol/mm^3^ medium). The concentration in the plastic (C_plastic_, nmol/mm^2^) depends on the amount in total plastic (A_plastic_, nmol) and the plastic surface area [P] (mm^2^ plastic/mm^3^ medium):

(1) $$C_{freeM}=\;\frac{C_{nominal}\times 1}{\left(1+K_{Serum}\times \left[S\right]\right)}$$

(2) $$C_{plastic}=\frac{A_{plastic}}{\left[P\right]}$$

The condition with Matrigel in the ECM channel included additionally the free concentration in the ECM (C_freeECM_, nmol/mm^3^) with the chemical amount in the ECM (A_ECM_, nmol), the ECM volume (V_ECM_, mm^3^), and the partitioning (K_ECMPr_ and K_Cell_, both µg/mm^3^) to ECM proteins ([ECMPr], µg protein/mm^3^ gel) and cell lipids ([C], µg/mm^3^ medium), respectively:

(3) $$C_{freeECM}=\frac{\frac{A_{ECM}}{V_{ECM}}\times 1}{\left(1+K_{ECMPr}\times \left[ECMPr\right]+\;K_{Cell}\times \left[C\right]\right)}$$

Next, we defined a set of differential equations that describe the bidirectional processes using mass transfer rate constants (k, in min^−1^) as illustrated in A. Changes in the chemical amount in the medium channel (Am_M_) and total plastic (A_plastic_) for conditions with and without serum were estimated by

(4) $$dAm_{M}=-C_{freeM}\times kMtoP+C_{plastic}\times kPtoM$$

(5) $$dA_{plastic}=C_{freeM}\times kMtoP-C_{plastic}\times kPtoM$$

dependent on the free concentration in medium (C_freeM_), concentration in total plastic (C_plastic_) and the rate constant between medium and plastic (kMtoP) and plastic to medium (kPtoM).

Changes in the condition with ECM were additionally estimated by

(6) $$dAm_{M}=-kMtoECM\times \left(C_{freeM}-C_{freeECM}\right)-C_{freeM}\times kMtoP+C_{plastic}\times kPtoM$$

(7) $$dA_{ECM}=kMtoECM\times \left(C_{freeM}-C_{freeECM}\right)-C_{freeECM}\times kMtoP+C_{plastic}\times kPtoM$$

where the changes in the medium channel also depend on the free amount in the ECM channel (C_freeECM_) and the rate constant between the medium and the ECM channel (kMtoECM).

To define the boundary conditions of the in silico mass distribution model, the following assumptions were made: (1) The equilibrium mass distribution of the chemical between compartments is linear, and described using the partition coefficient, K with logP and first-order rate constants (kMtoP, kPtoM, kMtoECM); (2) The chemical equilibrium with serum is instantaneous; (3) The bidirectional mass transfer between the medium and ECM channel (kMtoECM) represents a simplified diffusion; (4) The exchange with plastic is the same in the medium and ECM channel, so no specific kECMtoP is defined; (5) Single, non-dividing hepatocyte-like cells in Matrigel; (6) Dynamic flow conditions with a single exposure at a non-saturable concentration; (7) Only chemical losses (Alost) due to evaporation and plastic sorption were considered (as done before by [23,25,28,30]), but not those caused by xenobiotic metabolism, active cell transport, or spontaneous degradation.

To compare our model predictions with the steady-state equilibrium model by Kramer [25]we determined the unbound PAH concentrations using the partition coefficients for serum and plastic with the same [S] and [P] parameters as the Organoplate:

(8) $$C_{free}=\frac{C_{nominal}}{1+K_{Serum}\cdot \left[S\right]+K_{plastic}\times [P])}$$

## 2. Materials and Methods

### 2.1. Test Chemicals, Device and Extra Cellular Matrix Hydrogel

The individual PAHs were purchased as powder (all from Sigma, Zwijndrecht, The Netherlands; Supplementary Table S1) and dissolved separately to prepare master stocks in dimethyl sulfoxide (DMSO, CAS 67-68-5, Acros Organics, Geel, Belgium) at 1 mg/mL. These stocks were then used to prepare working dilutions and analytical standards. The MPS device employed is a 2-lane Organoplate^®^ (Mimetas B.V., Leiden, The Netherlands), which features a 384-well plate format comprising 96 culture chamber chips made from polystyrene, each with a perfusion channel and an ECM channel that are separated by a PhaseGuide™ (100 × 55 μm, w × h; see cross-section in A) made from a permanent dry film resist material. The perfusion channel measures 220 μm in height and 300 μm in width, with a length of 7 mm, while the ECM channel is 220 μm high, 500 μm wide, and 14.5 mm long. Perfusion is gravity driven using the Organoflow^®^ S device at the recommended 7° rocking angle, resulting in intermittent shear stress between 0 and 1.41 dyne/cm^2^. The Organoplate relies on ECM hydrogel for cell seeding; therefore, we selected for the third condition (Figure 1D) growth factor-reduced, phenol-red-free Matrigel (Corning, Bedford, MA, USA) because it is recognised as a primary tissue scaffold material suitable for various cell types with a relatively high protein content of 9.06 mg/mL [31].

### 2.2. Optimising Recovery and Extraction Protocols

As an initial step, we determined and refined the chemical recovery for bare (no serum) and 2% serum supplemented medium, Matrigel, and polystyrene plastic in a static environment. To develop an efficient liquid–liquid extraction protocol for the PAHs, we tested various solvents (e.g., DMSO, acetonitrile, isohexane, and n-hexane; all from Sigma) and combined them with different acids (hydrocholic acid, formic acid) to denature proteins, efficiently recover dissolved PAHs, and enable direct GC-MS/MS analysis.

The most successful contender, n-hexane with formic acid, was ultimately used. As part of the preparation, extraction hexane was prepared with five deuterated PAHs in hexane to serve as an internal standard that corrected for solvent evaporation during exposure and measurement (Dr. Ehrenstorfer, Augsburg, Germany; PAH-Mix 24, 10 µg/mL; from this point onward, ISTD-hex). Next, the individual PAH stocks in DMSO were diluted in mixture to a 1 mM working stock (0.1 mg/mL), which was used to determine the initial concentration (C_0_) control, prepare GC-MS analytical standards and to spike bare culture medium (final concentration 3–5 µM, see Supplementary Table S1 for C_0_). Then, the spiked bare culture medium was used to prepare serum conditions with 2% foetal calf serum (FCS, Thermo Fisher Scientific, Landsmeer, The Netherlands).

First, the initial concentration was determined by immediately recovering the PAHs from bare (no serum) medium before exposure (t = 0) to plastic and Matrigel, and before preparing the 2% serum supplemented medium. To do so, 80 µL of bare exposure media was transferred to amber autosampler glass vials with wide-mouth inserts containing ISTD-hex in a 1:1 ratio for the subsequent extraction step (see below). Second, 2% serum-supplemented medium was immediately recovered after preparation by transferring 80 µL to an amber autosampler glass vial with a wide-mouth insert containing 160 µL of ISTD-hex with 13 µL of formic acid for the subsequent extraction step. Third, 20 µL of Matrigel was polymerised in amber autosampler glass vials for 15 min at 37 °C and then covered with 200 µL of spiked bare medium for a 2 h exposure. After exposure, the covering medium was transferred to a wide-mouth insert containing ISTD-hex in a 1:1 ratio. Subsequently, the vial with Matrigel was rinsed with PBS, which was discarded, and 20 µL of RLT Buffer(Qiagen, Hilden, Germany) was added to lyse the Matrigel. Then, 50 µL of PBS and 20 µL of formic acid were added to the glass vial, vortexed, and then 320 µL of ISTD-hex was added for the subsequent extraction step.

The final extraction procedure step for medium, serum-supplemented medium, and Matrigel in ISTD-hex included vortexing for 2 h to maximise PAH recovery, centrifuging for 2 min at 1000 RPM to remove homogenate and obtain a clear hexane-aqueous layer. Finally, 50 µL of hexane was transferred into a fresh autosampler vial with a wide-mouth insert for direct GC-MS/MS analysis.

For plastic extraction, a 24-well flat bottom microtiter plate was covered with 250 µL of spiked bare medium and exposed for 2 h on a plate shaker. To minimise evaporation and light exposure, the well plate was covered with aluminium foil and wrapped with parafilm. After exposure, the bare medium was collected for 1:1 extraction (see steps above) and the remaining media were removed until the well was dry. A sample of 250 µL ISTD-hex was added 3× for 15 min to extract plastic-bound PAH and collected in an amber autosampler glass vial for direct measurement. During repeated ISTD-hex washouts, the well plate was covered with aluminium foil and wrapped in Parafilm to reduce evaporation before being returned to the vortex.

To minimise non-specific binding to plastic-made laboratory equipment, we kept plastic-contact times as low as possible during pipetting and used only glassware for stock and sample handling and storage.

### 2.3. Experimental Determination of Time-Dependent Chemical Mass Distribution on the 2-Lane Organoplate

To analytically determine the chemical mass distribution on the chip at the six selected time points (0.5, 1, 2, 4, 8, and 16 h) in the absence and presence of serum, as well as with Matrigel without serum, the above described extraction protocols were followed. ISTD-hex and working stocks were freshly prepared for the C_0_ control, GCMS standards and exposure media. The chemicals were extracted from the matrices immediately after sampling (Figure 1C).

In brief, the first two exposure conditions with bare and 2% FCS aimed to estimate the impact of serum, whereas the third condition was intended to assess the impact of Matrigel (Figure 1D) on the mass distribution. In each condition, 50 µL of spiked (supplemented) medium was added to the medium channel inlet and outlets (Figure 1C). For the third condition, the ECM channel was first filled with 2 µL of Matrigel, which polymerised for 15 min in the incubator, and then spiked bare (no serum) medium was added. After exposure, medium samples were collected for extraction (see protocol above), and exposed chips were briefly rinsed under gradient flow with a total of 50 µL PBS to remove medium residues. In the Matrigel condition, the gel was lysed after the PBS wash, also under gradient-driven flow, with a total of 50 µL of RLT Buffer. The lysate was then collected from the chip with a total of 50 µL PBS via gradient-driven flow and transferred for extraction (see protocol above).

As the final step in all conditions, the PAHs were extracted from the complete inside polystyrene plastic by adding 3× 110 µL ISTD-hex for 10 min in a volume gradient. To reduce evaporation during extraction, the plate was covered with tin foil and wrapped in parafilm during the repeated steps. The total ISTD-hex volume was collected in autosampler vials for direct GC-MS/MS measurement.

### 2.4. Chemical Analysis with GC-MS/MS

Quantification of PAHs in medium, plastic, and Matrigel was carried out using a Shimadzu GC-MS-TQ8040 tandem mass spectrometer in multiple reaction monitoring modes, Shimadzu AOC-20i Plus Auto-Injector, and Shimadzu GCMS solution workstation software (Version 4.52, Shimadzu, Kyoto, Japan). Analytes were separated with a Shimadzu SH-I-5Sil MS Column (0.25 mm, 0.25 μm, 30 m; Part Number: 221-75954-30). The flow rate was set to 1 mL/min, with hydrogen as the carrier gas (generated by a Peak Precision Hydrogen Trace 500 Hydrogen Generator, Düren, Germany). The column oven temperature was programmed as follows: an initial temperature of 80 °C, increased to 320 °C at 25 °C/min, and then held for 3 min. The total runtime was 16 min. The injection port temperature was maintained at 280 °C, and the injection volume was 1 μL in splitless mode. The ion source and interface temperatures were 280 °C and 320 °C, respectively. The limits of detection and quantification for the analytes were determined by analysing a measurement range of 1.5 nM to 10 µM in n-hexane, which also served as the calibration curve for quantifying the unknown samples.

## 3. Results and Discussion

### 3.1. Extraction Efficiency of PAHs from Different In Vitro Compartments

Before performing the exposure experiments on the Organoplate MPS, we developed an extraction protocol to maximise the recovery of the five PAHs from medium, serum, ECM, and polystyrene plastic. To achieve this, we tested various extraction solvents, including DMSO, acetonitrile, isohexane, and n-hexane, based on their polarity and volatility, and their potential for polystyrene plastic degradation over time. Of all the solvents, n-hexane most effectively separated all PAHs from the different compartments and allowed us to perform direct GCMS measurement, but it was also the most volatile and aggressive towards the plastic, necessitating us to moderate the extraction time from plastic.

**Figure 2:**
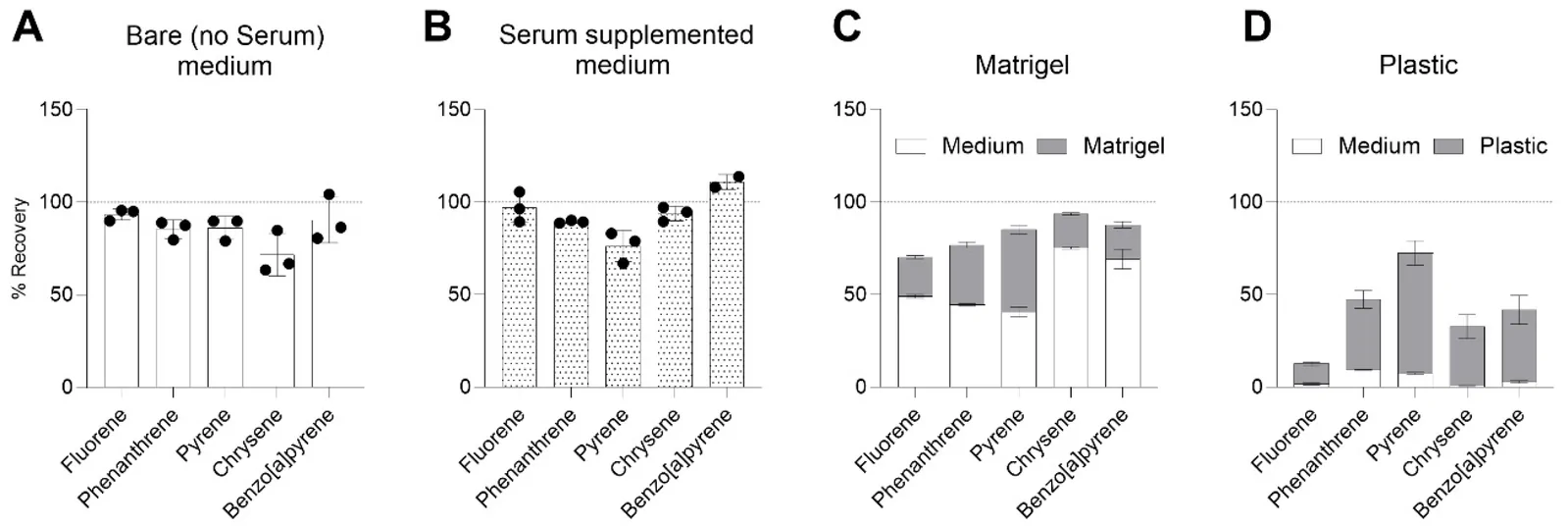
Extraction efficiency (%) from medium, Matrigel and plastic for fluorene, phenanthrene, pyrene, chrysene and benzo[a]pyrene (b[a]p) in static environment. (**A**) Recovery from bare (no serum) medium prepared in autosampler vial (n = 3), (**B**) recovery from 2% foetal calf serum-supplemented medium prepared in autosampler vial (n = 3), (**C**) recovery from extra cellular matrix hydrogel Matrigel prepared in an autosampler vial after two hours exposure with bare (no serum) medium (n = 3) and (**D**) recovery from polystyrene well-plate plastic after two hours exposure with bare (no serum) medium (n = 3). The data are reported as mean ± SD relative to the nominal amount added (t = 0).

Figure 2 displays the extraction efficiency from the different in vitro compartments after exposure in a static environment, where medium samples were prepared in glass vials and extracted immediately, while both Matrigel and polystyrene plastic samples were recovered after 2 h of exposure. In the no serum medium condition, approximately 71–93% of each chemical was recovered, although the variability increased slightly with hydrophobicity (Figure 2A). In the presence of 2% FCS, 76–110% of the chemicals were recoverable, with no apparent correlation to the hydrophobicity but a clear contribution of solvent evaporation [32]. For Matrigel, the overall recovery increased with hydrophobicity, starting at 70% for fluorene up to 93% for chrysene, whereas most chemical was recovered from the medium for all PAHs. Plastic recovery was generally low, even with the most aggressive extraction solvent, except for fluorene. The low recoveries of fluorene and to a lesser extent phenanthrene from Matrigel and plastic after 2 h of exposure were consistent with our expectation, as both are more volatile due to their fewer aromatic rings [28]. In contrast, chrysene and b[a]p have the highest lipophilicity among the tested PAHs, increasing their likelihood of adsorption to plastic, which partly explains their low recovery (45% and 42%) from that compartment [28,33]. Pyrene was included for its moderate volatility and lipophilicity, demonstrating therefore also the most moderate recovery in all compartments.

### 3.2. Mass Distribution of PAHs on the Organoplate Without Cells

**Figure 3:**
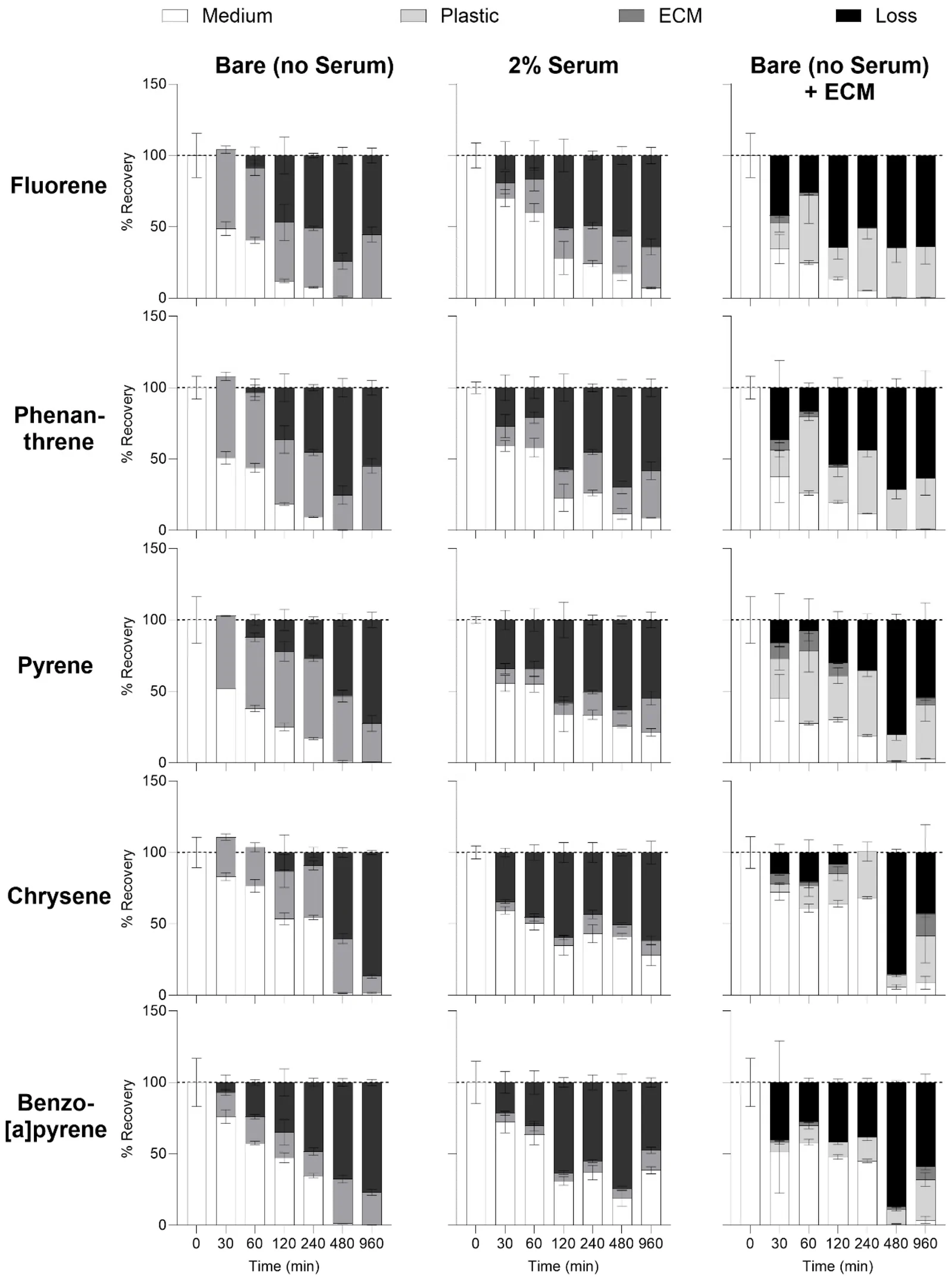
Mass distribution of the five polycyclic aromatic hydrocarbons in the three experimental conditions: bare (no serum) medium, 2% serum, bare (no serum) with ECM on the Organoplate MPS. Relative distribution in (supplemented) medium (white bar), total polystyrene plastic (light grey bar), Matrigel extracellular matrix (ECM) hydrogel (dark grey bar) and loss (black bar) after a single PAH mix exposure at t = 0. The data are reported as mean ± SD relative to the nominal amount (t = 0) from five replicates (n = 5) per time point.

On the Organoplate, the mass distribution of the PAH mixture was measured in (serum supplemented) medium, total plastic, and, if present, ECM after 0, 0.5, 1, 2, 4, 8, and 16 h to understand the time-dependent profile and to estimate losses due to either evaporation or plastic absorption (Figure 3). First, the bare (no serum) condition was assessed to estimate the impact of plastic binding in the absence of serum. Between 1 and 8 h of exposure, a clear trend was observed for all PAHs, except chrysene, showing a steady chemical decrease in the medium compartment with an increase in the total plastic, as well as a steep rise in losses. However, we did not observe equilibrium because of increasing losses, particularly at the final sampling time point (16 h), when no detectable amounts remained in the medium and, surprisingly, even fewer in the plastic for all PAHs. Although the culture vessel geometry of the static 96-well plate differs from the Organoplate, we observed, as expected, higher losses for fluorene, phenanthrene, and b[a]p from two hours onward, due to their physicochemical properties. More specifically, fluorene and phenanthrene are both less lipophilic and more volatile than the other tested PAHs, which causes them to evaporate rather than bind to plastic, even when the plate is covered with parafilm and aluminium foil during the experiments [30,34]. Based on the Organoplate design, which employs a 384-well plate format with a relatively small headspace compared to a 96-well plate (Figure 2), we did not expect sufficient surface area for evaporation; however, the continuously moving medium appeared to accelerate the process. In contrast, b[a]p and to a lesser extent chrysene, remained in the plastic despite multiple washing steps to extract them. Indeed, the Organoplate is a largely closed system consisting of polystyrene plastic microchannels that enclose the medium, thereby increasing the medium-to-plastic surface area to which the (highly lipophilic) PAHs can bind, but also increasing the surface area from which they must be extracted. Consequently, we decided to treat the losses as a given for all PAHs, given the technical feasibility, and accounted for them accordingly in the in silico mass balance model.

Next (Figure 3), the serum condition was analysed to estimate the impact of serum proteins on the mass distribution. Interestingly, a trend toward equilibrium in the medium compartment was observed for all PAHs after approximately 2 h, although the amounts recovered varied. Most amounts remained in the medium compartment for pyrene, chrysene, and b[a]p (~45%). In contrast, approximately only 10% of the total amount remained in the medium for fluorene and phenanthrene, likely due to their lower lipophilicity and, consequently, lower binding affinity. The largest differences were observed in the amounts recovered from plastic. Whereas fluorene and phenanthrene had the highest recovery from plastic, lower amounts of chrysene and b[a]p were recoverable, leading to large chemical losses (50–60%), likely due to an increased plastic sorption with time [20,28]. Most notably, compared to the bare condition, equilibrium was reached even for the losses because the PAHs were bound and unable to move compartments. More specifically, serum is a dominant binding component, resulting in a high chemical fraction remaining in the medium compartment; however, this process is saturable, leading to the free fraction eventually binding to plastic and reaching equilibrium, with lower losses [27]. Nevertheless, we also need to emphasise that although serum is a dominant binding component, the losses remain very high and are still comparable to our observations in the bare condition.

Lastly (Figure 3), we analysed the condition in which Matrigel was exposed to bare (no serum) medium to assess the impact of the ECM on mass distribution. Notably, there was substantial variation in recoverable amounts across compartments and for each PAH, with no clear indication of equilibrium over time. While the amounts of fluorene, phenanthrene and pyrene decreased steadily in the medium, the amounts in plastic increased only slowly, primarily due to substantial losses. In contrast, more than half of chrysene and b[a]p remained in the medium compartment during the first four hours, although about half was lost after eight hours. Remarkably, for all PAHs, low amounts were detectable in the Matrigel within the first two hours, and after that only for chrysene and b[a]p, as they are more lipophilic. Indeed, ECM protein binding and the small volume of Matrigel used may have reduced the recoverable amount of all PAHs, resulting in levels below the limit of quantification, which were ultimately considered losses [11]. Compared with the bare and serum conditions, there were, in general, higher losses, lower amounts in the medium early on, and a markedly lower recovery from plastic, with greater variability. Considering that the cell seeding in the Organoplate relies on the use of ECM to establish the culture environment, these large measurement variabilities would likely also influence model robustness for other assays [35,36].

### 3.3. Modelled Mass Balance Kinetics and Bound Fractions

**Figure 4:**
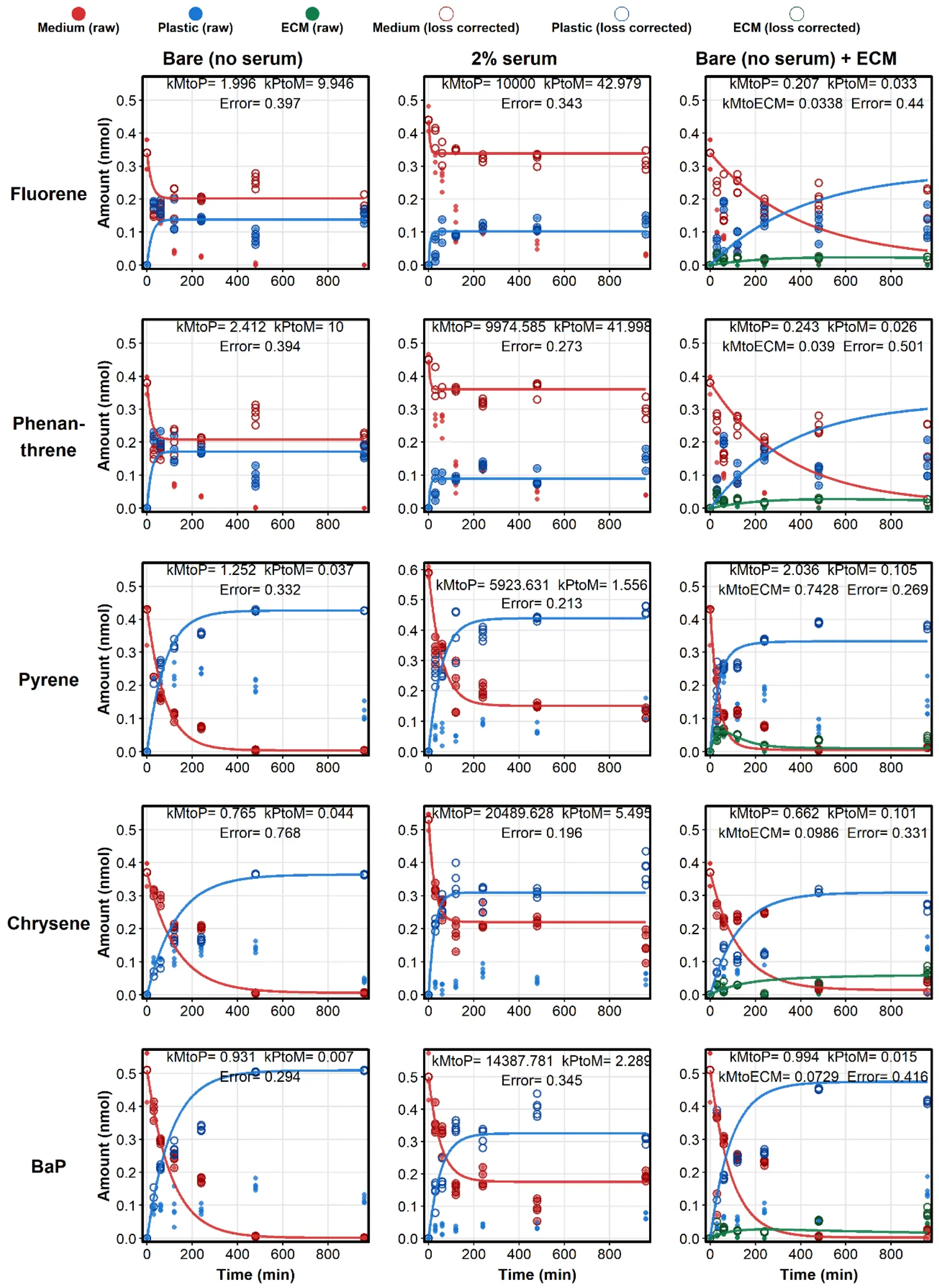
Mass balance kinetics of the five polycyclic aromatic hydrocarbons in the three experimental conditions: bare (no serum), 2% foetal calf serum, bare (no serum) with Matrigel ECM hydrogel. Plots show the amounts (in nmol) in medium (red), polystyrene plastic (blue) and ECM (green) over 960 min. Uncorrected experimental raw values (red/blue/green full circles) are depicted as reference, together with evaporation/plastic absorption corrected experimental data (red/blue/green hollow circles). The prediction curve (red/blue/green) was based on loss-corrected data, with each data point displaying a technical replicate (n = 5). Plots include the generated best fit for the rate constant to plastic (kMtoP), from plastic to medium (kPtoM), and from medium to the ECM channel (kMtoECM) with the prediction error.

To determine the bound and free PAH fractions in the compartments, we modelled the time-dependent mass balance kinetics using both uncorrected and loss-corrected experimental mass distribution data for all five PAHs in the three conditions. The uncorrected mass balance simulation (Supplementary Figure S1) represents the observed reality, whereas the loss correction aimed to emulate an “ideal system”; as we have observed, there were considerable losses due to experimental evaporation and plastic absorption in the experimental data (Figure 3). This correction allowed us to extrapolate what the bound/free amounts would be if the entire nominal dose were available in the system, which is essential for QIVIVE. Therefore, we included chemical-, mass distribution-, and time-specific corrections to the medium and plastic compartments (hollow circles in Figure 4) in the error function prior to running the ordinary differential equations (ODE) optimisation scripts in R studio. In more detail, we corrected for evaporation of fluorene and phenanthrene by adding the full lost amount back to the medium compartment at each time point. Similarly, pyrene, chrysene and b[a]p were corrected for plastic sorption by adding the full lost amount back to the plastic compartment. The loss correction in the ECM condition used the same correction logic, but added instead of 100%, only 90% chemical back to the corresponding compartments and 10% to the ECM, as we also assumed a non-recoverable fraction there. Although this correction procedure is highly model assumption-dependent and lacks external validation of the losses, this approach allowed us to account for the entire (nominal) dose given the varied physicochemical properties of the tested PAHs and technical constraints of the device. Without this correction, the ODE model assumes by default complete binding to the most predominant compartment (serum or plastic), as can be seen in the uncorrected simulations (Supplementary Figure S1) and predictions (Table 1, Table 2 and Table 3). The performed sensitivity analysis (Supplementary Figures S2–S5) showed that the predicted free fraction was primarily controlled by the balance between medium-to-plastic and plastic-to-medium exchange in both the corrected and loss-corrected model predictions. However, although the absolute parameter estimates and predicted fractions differed between the two predictions, the overall dependence of the free fraction on the balance between forward and reverse exchange remained consistent.

Figure 4 displays, for all three experimental conditions, the raw (uncorrected) and corrected data, the prediction curves over 16 h of exposure with a non-saturable concentration, and the best-fitted rate constants with their prediction errors. First, the no serum condition prediction curves suggest a correlation between lipophilicity and time to reach equilibrium: while fluorene and phenanthrene take approximately 60 min, b[a]p takes ten times as long (~600 min). However, the rate constants, defined as the time the PAHs take to move from one compartment to another, do not necessarily correlate with the lipophilicity. While fluorene and phenanthrene have the largest kMtoP, meaning the fastest binding to the plastic, they also have the fastest reversibility of plastic binding (kPtoM). Considering the lipophilicity of the remaining three PAHs, we expected that kMtoP would increase with the amount of aromatic rings, whereas kPtoM would decrease; however, pyrene deviated from this pattern, likely due to the 100% plastic correction applied. Table 1 shows for all PAHs the corresponding experimentally recovered fractions together with the modelled bound fractions in the medium and plastic compartments using both, the raw (uncorrected, Supplementary Figure S1) and corrected data. Using the uncorrected data, the model predicts that most of the PAH mass is bound to the plastic compartment, even though we exposed the system to a non-saturable amount. This reflects a constraint scenario in which the model assumes dominant plastic binding, based on the negligible measured amount in the medium (~1%). By contrast, the loss-corrected data model yields drastically different predictions for fluorene and phenanthrene, indicating that only about half of each is bound to the plastic, based on the larger amount in the medium and their logP (lipophilicity). However, we acknowledge that adding an air compartment that accounts for evaporation is an improvement to the model’s robustness. This can be done either through an experimental determination [37], fitting another rate constant or by using the air–water partition coefficients with Henry’s Law constant to improve the model’s robustness [17,38,39]. However, for us this would also have challenged the technical feasibility of deriving data with our MPS device, increased the mass balance model complexity, and thereby extended beyond the scope of this exploratory study.

Next, in Figure 4, the 2% serum condition shows rapid equilibrium prediction curves (max. ~200 min) for all PAHs compared to the bare (no serum) condition, which is also reflected in the large rate constant kMtoP (1000–20,500). The large kMtoP results from the model assuming an instant partitioning to serum, yielding only a small fraction available to move compartments, especially for chrysene and b[a]p. Table 2 underpins the observation, as the order of rate constants correlates with the bound fraction to serum but not with lipophilicity (logP). Indeed, Table 2 confirms, in comparison with Table 1, that serum proteins are a major sink for chemical partitioning. However, we did not experimentally determine the unbound fraction in the recovered medium, as it is resource-intensive and the MPS device only provides small media volumes for analysis (100 µL) [5,40]. Nevertheless, we assume that the recovered chemical fraction in medium was serum bound, as this would be within the dominant binding affinity expectation [16,27]. Interestingly, the biggest differences between the two ODE models were observed only for fluorene and phenanthrene, while the other PAH outputs differed by 1–9%. More specifically, the raw (uncorrected) data indicate that the largest amount of fluorene and phenanthrene is bound to plastic (~91 and 93%, respectively), whereas the loss-corrected data indicate that the largest amount is bound to serum (77 and 80%, respectively). Furthermore, contrary to our expectations, chrysene showed higher serum binding than b[a]p (~51/42 vs. 34/35%, respectively), although b[a]p was our most lipophilic tested PAH. Also, contrasting with our expectation, was the predicted larger plastic binding for pyrene (~78/74%, respectively), rather than b[a]p (~66/65%, respectively), particularly given the predicted lower serum binding. Yet, there are uncertainties as these predictions are based on experimental data with some considerable variability, particularly for chrysene.

Lastly, in Figure 4, the ECM condition with bare (no serum) medium also shows large differences across the PAHs in the prediction curves and fitted rate constants, with pyrene being the most pronounced against our expectations. More specifically, for all PAHs but fluorene, the presence of ECM slows down the kMtoP compared to the other conditions. While fluorene reaches equilibrium within ~200 min, chrysene and b[a]p require approximately 500 min, and both fluorene and phenanthrene reach only an apparent equilibrium at approximately 1000 min; however, we cannot confirm this observation because the last sample was taken after 960 min. Additionally, only fluorene shows first an increase and then decrease in the ECM, which likely acted as an intermediate binding compartment due to the presence of proteins to bind to [29,41]. Yet after ~120 min, the plastic binding predominates again for pyrene, resulting in a shift from the ECM to the plastic compartment. Apart from pyrene breaking the correlation pattern, we observed that the kMtoP rate increased with lipophilicity (logP), whereas the kPtoM rate was generally lower for fluorene and phenanthrene, indicating a lower plastic-binding reversibility. The model predictions in Table 3 show that, unlike the bare (no serum) and 2% conditions, the loss-corrected ODE model now attributes more chemical bound to plastic for fluorene and phenanthrene, likely due to a longer retention time in the medium channel, combined with the media movement as the ECM is hampering the chemical distribution across the chip. The most contrasting finding between the two ODE models are the fractions bound to the ECM proteins, as we added for all PAHs 10% of the lost amount back to the ECM. Despite recovering a considerable amount for pyrene, chrysene and b[a]p, not all is predicted to be bound, except for chrysene, which behaved similarly in the serum condition. Ideally, further studies assess the partitioning coefficient to ECM proteins as we used the value derived in the study by Zhang and colleagues [29]. More specifically, Zhang and colleagues quantitatively characterised small molecule binding to Matrigel but also pointed out approximate trends with the logP and also data scattering for larger molecules. This is not surprising, as Matrigel varies by the batch in composition and therefore in binding sites. A comparative study as previously suggested by us, can help optimise the availability to the cells [42].

### 3.4. Fractions Unbound

Using the steady-state partition equilibrium model by Kramer and colleagues ([25], Equation (8)) and our time-dependent mass balance model, we derived and compared the free fractions that would be available to reach cells in an NGRA application (Table 4). Additionally, we theoretically extrapolated our findings using the fitted rate constants (Figure 4) to a typical application using the Organoplate: 10% FCS and Matrigel with 20,000 (HepaRG) cells in the ECM channel [36,42,43,44,45,46].

In the bare (no serum) condition, a considerable free fraction (11.8–0.23%, decreasing with increasing logP) is available when applying the Kramer model. An even greater fraction (~50%) is available when using our “ideal system” loss-corrected model, but only for fluorene and phenanthrene. Given that most of the fluorene and phenanthrene have actually evaporated in our experiments performed at room temperature, the free fraction derived from the uncorrected data (<1%) is potentially more accurate because evaporation is a real concern in any open cell-culture system, as reported by Rude [30] and Birch [34]. When comparing the 2% serum condition results, Kramers steady-state equilibrium model is more optimistic (10.64–0.22%), as our time-dependent mass balance model predicts barely any free fraction available (0.03–0%), with the majority bound to either serum or plastic. Not surprisingly, when extrapolating to 10% serum in the medium, even less PAH is unbound (0.01–0%). Given that this condition closely resembles an actual culture setup in this and similarly designed MPS, our findings suggest that the combination of this device design and low, below saturation doses may introduce uncertainty in the assessment of low-dose exposure to lipophilic compounds, particularly when investigating molecular mechanisms underlying toxicity [22,47]. Additionally, our findings also underpin the work by Nicol [5] and Henneberger [16], who highlight the misconception that nominal-to-nominal extrapolation is always a valid surrogate for risk assessment. A nominal-to-nominal approach often works for compounds where in vitro and in vivo free fractions are similar, based on 6–8% human plasma protein [5,16]. However, it fails for chemicals like PAHs due to their volatility, tendency to bind to plastic and ECM proteins, resulting in losses from the media [18]. This raises the question of whether a reliable point of departure can even be determined at high doses. If increasing the dose is necessary just to counteract these losses and elicit a cellular response, then the point of departure reflects laboratory limitation rather than an actual biological threshold, leading to inaccurate QIVIVE. Ideally, future studies should build on this descriptive and explanatory model by externally validating its predictive performance using independent experimental data from other groups of hydrophobic substances (e.g., polychlorinated biphenyls, phthalates) and, where appropriate, other MPS designs. This can help determine how far this model’s applicability domain can be generalised beyond PAHs and identify the system- and chemical-specific parameters required to predict chemical distribution across different hydrophobic substances and MPS platforms.

## 4. Conclusions

In conclusion, the mass distribution analysis, together with time-dependent mass balance modelling, provides a solid foundation for understanding the in vitro chemical kinetics in the Organoplate 2-lane MPS. Our mass distribution analysis showed significant losses over time due to evaporation and plastic sorption, driven by the MPS system characteristics: a high medium-to-plastic surface ratio and a continuous medium movement. Consequently, we used loss-corrected experimental data to fit the rate constants, which we then used to predict the fractions of bound and free PAHs. Our model predictions indicated that, under any condition, a large fraction is bound to any model compartments, especially in the presence of serum. Even at only 2% serum, less than 0.5% of the nominally added concentration was freely available. Additionally, we found that Matrigel in the ECM channel slows the overall mass balance kinetics, leading to a delayed partition equilibrium (between 8 and 16 h). These findings emphasise the limitations of ECM-dependent MPS systems, as the combination of flow with serum-supplemented medium leaves only a very low unbound fraction (<0.1%) available to reach the cells to be able to determine cellular effects in an actual NGRA application.

## Figures and Tables

**Table 1:** Measured and (uncorrected/loss corrected) modelled bound fractions in medium and plastic relative to the nominally added amount. ODE = ordinary differential equation model.

Bare (No Serum) Model at Equilibrium (960 min)						
	Measured				Predicted with ODE Model (No Correction)	Predicted with ODE Model (Loss Corrected)
Chemical	Recovered Fraction Medium in %	±SD	Recovered Fraction Plastic in %	±SD	Bound Fraction to Plastic in %	Bound Fraction to Plastic in %
Fluorene	0.07	0.05	44.6	5.23	99.41	40.69
Phenanthrene	0	0	45.2	5.12	99.82	45.19
Pyrene	0.93	0.12	29.4	6.01	99.15	99.15
Chrysene	1.7	0.38	11.7	1.45	98.28	98.28
B[a]p	0.25	0.18	22.8	2.07	99.75	99.75

**Table 2:** Measured and (uncorrected/loss corrected) modelled bound fractions in 2% serum-supplemented medium and plastic relative to the nominally added amount. ODE = ordinary differential equation model.

The 2% Serum Model at Equilibrium (960 min)								
	Measured				Predicted with ODE Model (No Correction)		Predicted with ODE Model (Loss Correction)	
Chemical	Recovered Fraction Medium in %	±SD	Recovered Fraction Plastic in %	±SD	Bound Fraction to Serum in %	Bound Fraction to Plastic in %	Bound Fraction to Serum in %	Bound Fraction to Plastic in %
Fluorene	7.2	0.63	28.7	5.6	7.21	92.79	76.76	23.21
Phenanthrene	8.84	0.14	33	6.16	8.9	91.09	80.01	19.96
Pyrene	21.4	2.7	24	4.84	21.8	78.41	25.6	74.39
Chrysene	28.26	7.45	10.1	2.91	50.85	49.14	41.59	58.4
B[a]p	38.5	2.4	14	2.16	34.13	65.86	35.01	64.99

**Table 3:** Measured and (uncorrected/loss corrected) modelled bound fractions in medium, plastic and Matrigel ECM relative to nominally added amount. ODE = ordinary differential equation model.

Bare (No Serum) with ECM Model at Equilibrium (960 min)										
	Measured						Predicted with ODE Model (No Correction)		Predicted with ODE Model (Loss Correction)	
Chemical	Recovered Fraction Medium in %	±SD	Recovered Fraction Plastic in %	±SD	Recovered Fraction ECM in %	±SD	Bound Fraction to Plastic in %	Bound Fraction to ECM in %	Bound Fraction to Plastic in %	Bound Fraction to ECM Protein in %
Fluorene	0.38	0.3	35.8	12.3	0	0	51.37	0	75.65	6.38
Phenanthrene	0.85	0.2	35.9	12	0	0	51.61	0	79.15	6.3
Pyrene	3.09	0.4	37.9	11.8	4.66	1.7	50.96	0.08	77.48	2.39
Chrysene	8.8	4.6	40.9	5	15.54	2.8	90.47	0.62	83.44	15.66
B[a]p	3.7	2.5	28.3	4.8	9	2.1	98.55	2.76	92.99	3.37

**Table 4:** Calculated and modelled unbound fractions relative to the nominal concentration added in three conditions: Bare (no serum), 2% and * 10% serum with ECM. * Theoretical extrapolation using the derived rate constants. ODE = ordinary differential equation model.

	Unbound Fractions (* Theoretical Extrapolation)						
	Bare (No Serum)			2% Serum			10% Serum with ECM *
	Steady-State Equilibrium Partition Model by Kramer et al. (2010) [25]	Predicted with No Loss-Corrected ODE Model	Predicted with Loss-Corrected ODE Model	Steady-State Equilibrium Model by Kramer [25]	Predicted with No Loss-Corrected ODE Model	Predicted with Loss-Corrected ODE Model	Predicted with ODE Model
Fluorene	11.98	0.59	59.31	10.64	0	0.03	0.01
Phenanthrene	9.36	0.18	54.81	8.51	0	0.02	0.01
Pyrene	3.61	0.85	0.85	3.48	0	0.01	0
Chrysene	0.38	1.72	1.72	0.38	0.01	0	0
B[a]p	0.23	0.25	0.25	0.22	0	0	0

## Data Availability

The data presented in this study is included in GitHub (https://github.com/nitsc005/Mass-Distribution, accessed on 13 July 2026). Further inquiries can be directed to the corresponding authors.

## Supplementary Materials

The following supporting information can be downloaded at: https://www.mdpi.com/article/10.3390/toxics14090837/s1, Supplementary Table S1: Properties and stock concentration of five polycyclic aromatic hydrocarbons tested; Supplementary Table S2: Gas chromatography mass spectrum parameters, limit of detection (LOD) and limit of quantification (LOQ) of 5 tested polycyclic aromatic hydrocarbons and deuterated internal standard polycyclic aromatic hydrocarbons; Supplementary Figure S1: Mass balance kinetics of the five polycyclic aromatic hydrocarbons in the three experimental conditions: bare (no serum), 2% fetal calf serum, bare (no serum) with Matrigel ECM hydrogel. Plots show the amounts (in nmol) in medium (red), plastic (blue) and ECM (green) over 960 min. Prediction curves (red/blue/green) were based on raw (uncorrected data, red/blue/green full circle) with each data point displaying a technical replicate (n = 5). Plots include the generated best fit for the rate constant to plastic (kMtoP) and from plastic to medium (kPtoM) and from medium to the ECM channel (kMtoECM) with the prediction error; Supplementary Figure S2: Predicted equilibrium fractions of the five polycyclic aromatic hydrocarbons in the three experimental conditions: bare (no serum), 2% fetal calf serum, bare (no serum) with Matrigel ECM hydrogel after 960 min. The plot was generated from 50 bootstrap datasets by resampling the five experimental replicates with replacement. The model was refitted to each bootstrap dataset, and 95% percentile confidence intervals were calculated from the equilibrium-fraction distributions. Bars show the best-fit predicted percentages of the nominal amount present as the free medium fraction, serum-bound fraction, plastic-bound fraction, or ECM-bound fraction, based on the raw/uncorrected data. Error bars represent 95% bootstrap confidence intervals; Supplementary Figure S3: Best-fit rate constants for the distribution of the five polycyclic aromatic hydrocarbons in the three experimental conditions: bare (no serum), 2% fetal calf serum, bare (no serum) with Matrigel ECM hydrogel after 960 min. The plot was generated from 50 bootstrap datasets by resampling the five experimental replicates with replacement. The model was refitted to each bootstrap dataset, and 95% percentile confidence intervals were calculated from the resulting parameter distributions. Bars show the estimated transfer-rate parameters obtained by fitting the compartmental model to the raw/uncorrected data: medium-to-plastic transfer ($k_{M\to P}$), plastic-to-medium transfer ($k_{P\to M}$), and, where applicable, medium-to-ECM transfer ($k_{M\to ECM}$). Values are expressed in ${min}^{-1}$ on a logarithmic scale. Error bars represent 95% percentile bootstrap confidence intervals based on 50 resampled datasets; Supplementary Figure S4: Predicted equilibrium fractions of the five polycyclic aromatic hydrocarbons in the three experimental conditions: bare (no serum), 2% fetal calf serum, bare (no serum) with Matrigel ECM hydrogel after 960 min. The plot was generated from 50 bootstrap datasets by resampling the five experimental replicates with replacement. The model was refitted to each bootstrap dataset, and 95% percentile confidence intervals were calculated from the resulting equilibrium-fraction distributions. Bars show the best-fit predicted percentages of the nominal amount present as the free medium fraction, serum-bound fraction, plastic-bound fraction, or ECM-bound fraction, based on the loss-corrected data. Error bars represent 95% bootstrap confidence intervals; Supplementary Figure S5: Best-fit rate constants for the distribution of the five polycyclic aromatic hydrocarbons in the three experimental conditions: bare (no serum), 2% fetal calf serum, bare (no serum) with Matrigel ECM hydrogel after 960 min. The plot was generated from 50 bootstrap datasets by resampling the five experimental replicates with replacement. The model was refitted to each bootstrap dataset, and 95% percentile confidence intervals were calculated from the resulting parameter and distributions. Bars show the estimated transfer-rate parameters obtained by fitting the compartmental model to the loss-corrected data: medium-to-plastic transfer ($k_{M\to P}$), plastic-to-medium transfer ($k_{P\to M}$), and, where applicable, medium-to-ECM transfer ($k_{M\to ECM}$). Values are expressed in ${min}^{-1}$ on a logarithmic scale. Error bars represent 95% percentile bootstrap confidence intervals based on 50 resampled datasets.
