# Supplementary material for: Chemical Distribution Kinetics of Five Polycyclic Aromatic Hydrocarbons in a Microphysiological System
Source: Toxics. 2026 Sep 21;14(9):837. doi: 10.3390/toxics14090837 (PMC13611211; doi:10.3390/toxics14090837)
Supplement: Supplementary file 1 [file toxics-14-00837-s001.zip › toxics-4468464-supplementary.pdf]

SUPPLEMENTAL MATERIALS

Chemical distribution kinetics of five polycyclic aromatic hydrocarbons in a microphysiological system

Katharina S. Nitsche[1], Susana Proença[2], Wouter Bakker[1], Paul Carmichael[1,3], Hans Bouwmeester[1] and Nynke I. Kramer[1]

1 Division of Toxicology, Wageningen University & Research, P.O. Box 8000,
6700 EA Wageningen, The Netherlands; hans.bouwmeester@wur.nl (H.B.)
2 esqLABS GmbH, 26683 Saterland, Germany
3 Unilever Safety and Environmental & Regulatory Science Group, Colworth Science Park, Sharnbrook, Bedfordshire MK44 1LQ, UK

Supplementary Table S1 Properties and stock concentration of five polycyclic aromatic hydrocarbons tested.

Compound	*LogP*	*Molecular Weight (g/mol)*	*CAS*	*C_0 in mix at time of experiment (µM)*
Fluorene	4.18	166.22	86-73-7	3.32
Phenanthrene	4.46	178.23	85-01-8	3.44
Pyrene	4.88	202.25	129-00-0	3.52
Chrysene	5.81	228.29	218-01-9	4.5
Benzo[a]pyrene	6.13	252.31	50-32-8	3.76

Supplementary Table S2 Gas chromatography mass spectrum parameters, limit of detection (LOD) and limit of quantification (LOQ) of 5 tested polycyclic aromatic hydrocarbons and deuterated internal standard polycyclic aromatic hydrocarbons

	Mode	Target Ion	Qualifier Ion	Retention time (min)	LOD (µM) in n-Hex	LOQ (µM) in n-Hex
Internal standards						
Acenapthtene-d10	SIM	164		6.0	-	-
Phenanthrene-d10	SIM	188		7.6	-	-
Chrysene-d12	SIM	240		10.4	-	-
Perylene-d12	SIM	264		11.9	-	-
Analytes						
Fluorene	SIM	166	165.00; 82.00	6.6	0.01 µM	0.02 µM
	MRM	166.00>165.00 (30V)	166.00>163.00 (34V)			
Phenanthrene	SIM	178	176.00; 152.00	7.6	0.04 µM	0.08 µM
	MRM	178.00>176.00 (34V)	178.00>152.00 (30V)			
Pyrene	SIM	202	200.00; 101.00	9.1	0.04 µM	0.08 µM
	MRM	202.00>200.00 (50V)	202.00>201.00 (30V)			
Chrysene	SIM	228	226.00; 114.00	10.5	0.04 µM	0.08 µM

	MRM	228.00>226.00 (38V)	228.00>224.00 (38V)			
Benzo[a]pyrene	SIM	252	250.00; 126.00	11.9	0.03 μM	0.08 μM
	MRM	252.00>250.00 (40V)	252.00>248.00 (40V)			

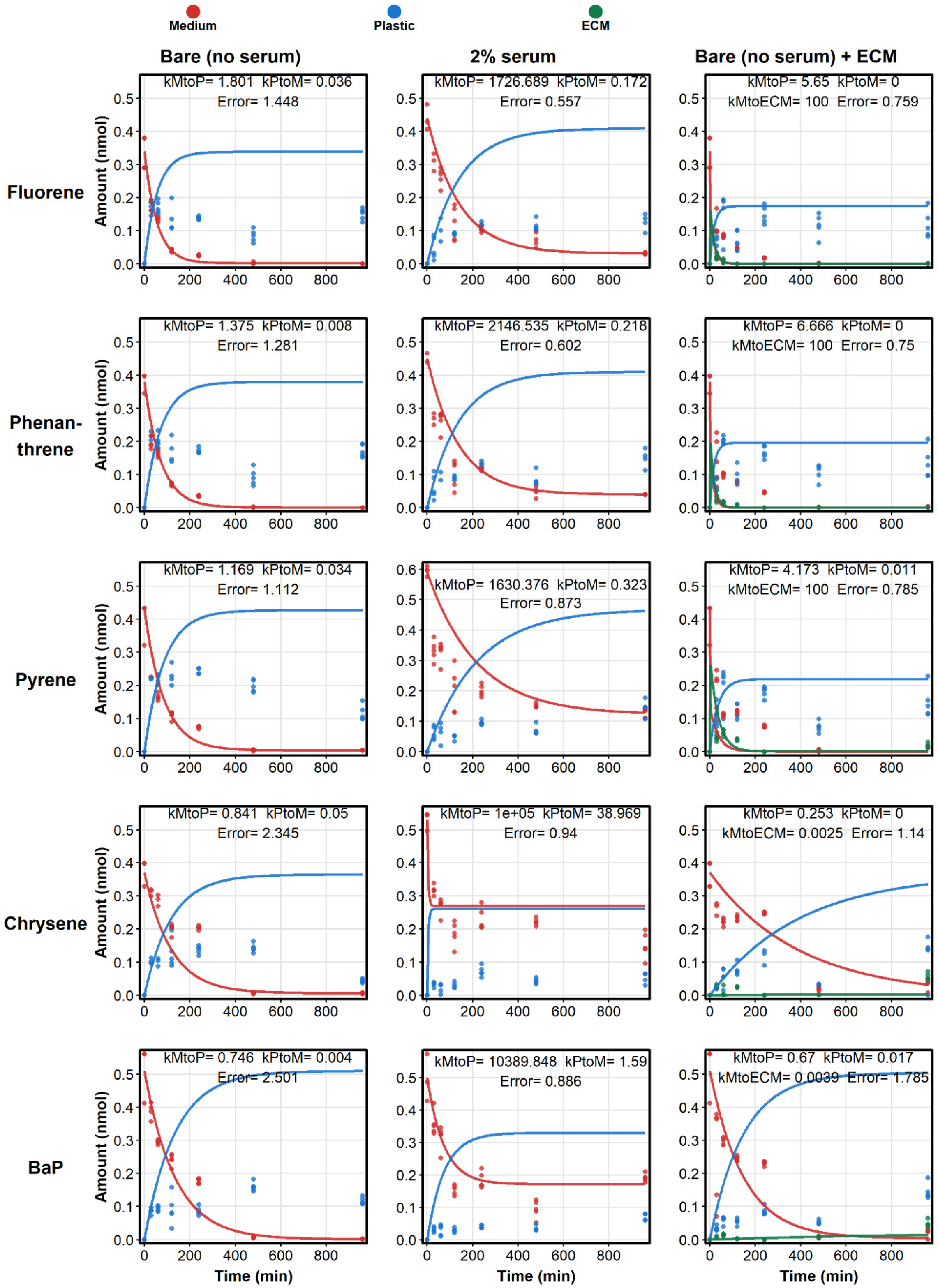

Supplementary Figure S1 Mass balance kinetics of the five polycyclic aromatic hydrocarbons in the three experimental conditions: bare (no serum), 2% fetal calf serum, bare (no serum) with Matrigel ECM hydrogel. Plots show the amounts (in nmol) in

medium (red), plastic (blue) and ECM (green) over 960 minutes. Prediction curves (red/blue/green) were based on raw (uncorrected data, red/blue/green full circle) with each data point displaying a technical replicate (n=5). Plots include the generated best fit for the rate constant to plastic (kMtoP) and from plastic to medium (kPtoM) and from medium to the ECM channel (kMtoECM) with the prediction error.

Methodology description sensitivity analysis

Model fitting stability and parameter identifiability were assessed by repeated optimisation from independent starting values and by evaluation of the objective function over a local parameter grid. For each polycyclic aromatic hydrocarbon and each experimental condition, model parameters were fit to time-course concentration data from five experimental replicate wells (medium, plastic, and, where applicable, ECM compartments). For each condition, the model was refit from 20 random starting points in log-parameter space, and all solutions within 5% of the minimum objective value were retained to characterise the range of kMtoP and kPtoM values obtained across fits. Parameter identifiability was further examined by evaluating the objective function over a local log-spaced grid of kMtoP and kPtoM values around the best-fit solution, and by performing a nonparametric bootstrap in which the five experimental replicates were resampled with replacement and the model refit to each resampled dataset. The correlation between the bootstrapped log-transformed rate constants was calculated to describe the degree of parameter trade-off.

Model code can be found at https://github.com/nitsc005/Mass-Distribution

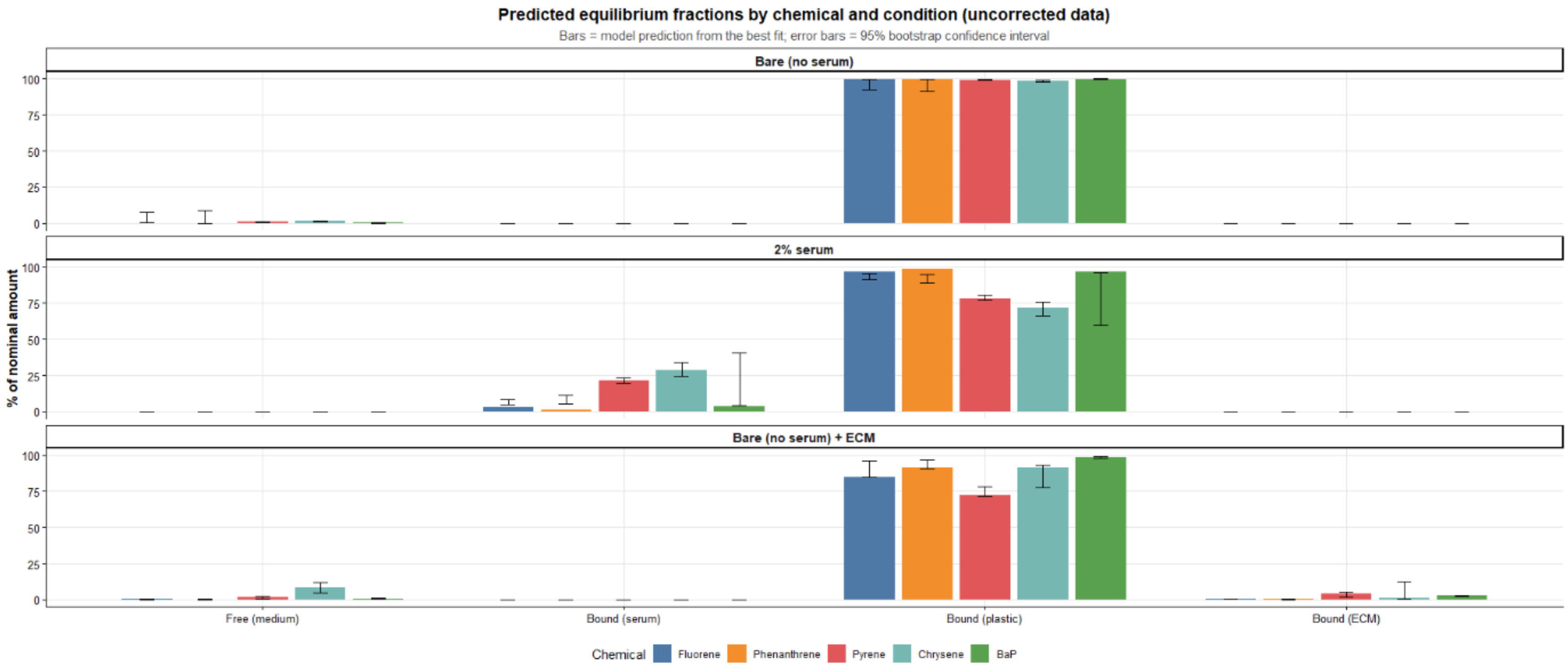

Supplementary Figure S2. Predicted equilibrium fractions of the five polycyclic aromatic hydrocarbons in the three experimental conditions: bare (no serum), 2% fetal calf serum, bare (no serum) with Matrigel ECM hydrogel after 960min. The plot was generated from 50 bootstrap datasets by resampling the five experimental replicates with replacement. The model was refitted to each bootstrap dataset, and 95% percentile confidence intervals were calculated from the equilibrium-fraction distributions. Bars show the best-fit predicted percentages of the nominal amount present as the free medium fraction, serum-bound fraction, plastic-bound fraction, or ECM-bound fraction, based on the raw/uncorrected data. Error bars represent 95% bootstrap confidence intervals.

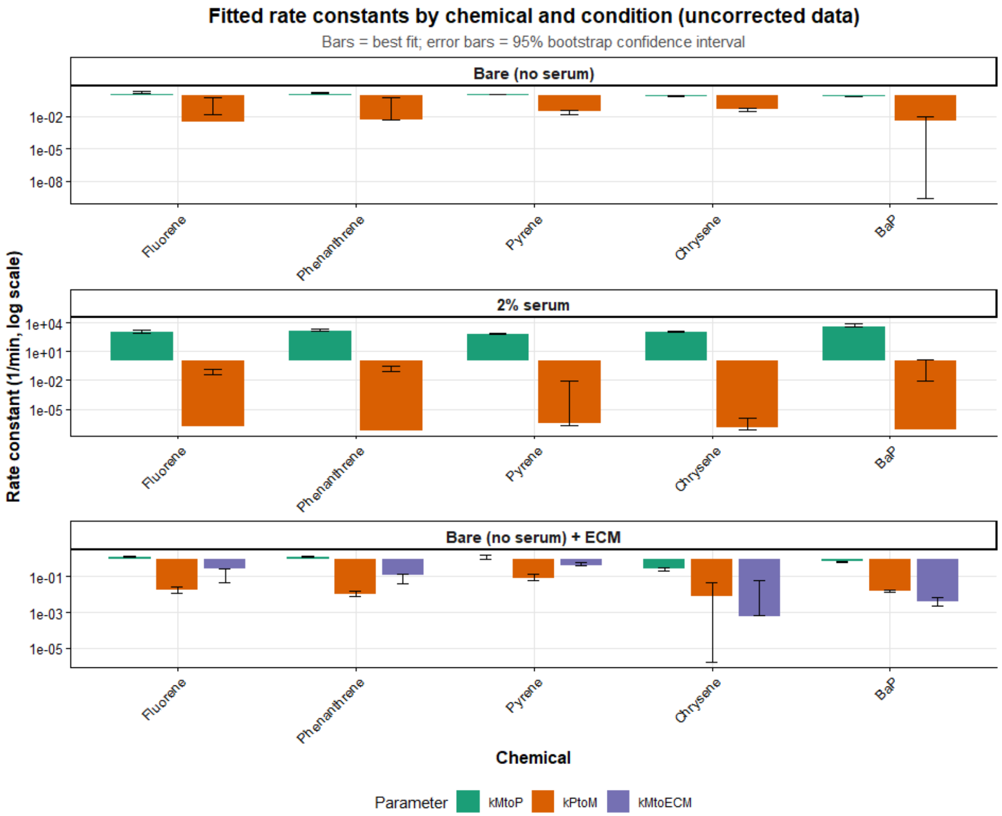

Supplementary Figure S3. Best-fit rate constants for the distribution of the five polycyclic aromatic hydrocarbons in the three experimental conditions: bare (no serum), 2% fetal calf serum, bare (no serum) with Matrigel ECM hydrogel after 960min. The plot was generated from 50 bootstrap datasets by resampling the five experimental replicates with replacement. The model was refitted to each bootstrap dataset, and 95% percentile confidence intervals were calculated from the resulting parameter distributions. Bars show the estimated transfer-rate parameters obtained by fitting the compartmental model to the raw/uncorrected data: medium-to-plastic transfer ($k_{M\rightarrow P}$), plastic-to-medium transfer ($k_{P\rightarrow M}$), and, where applicable, medium-to-ECM transfer ($k_{M\rightarrow ECM}$). Values are expressed in min^{-1} on a logarithmic scale. Error bars represent 95% percentile bootstrap confidence intervals based on 50 resampled datasets.

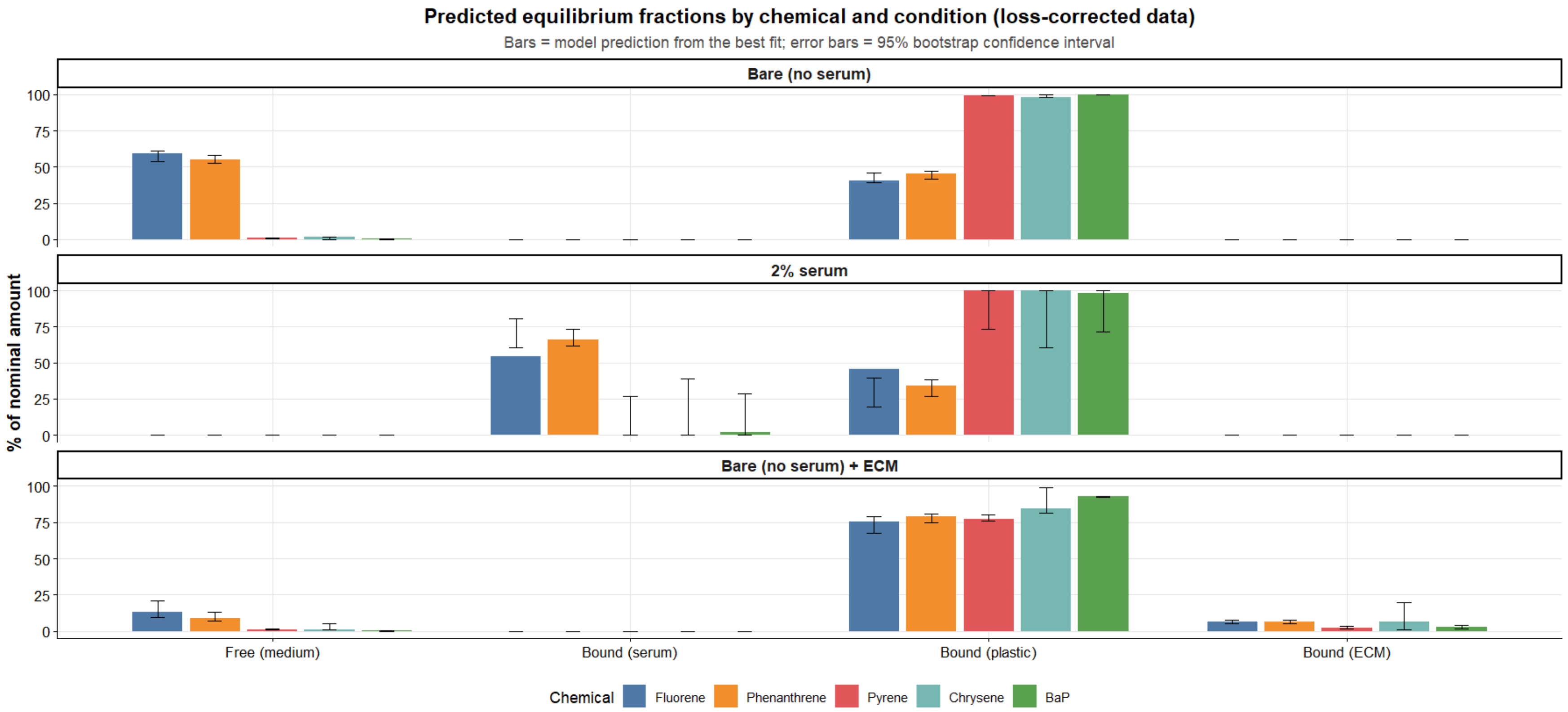

Supplementary Figure S4. Predicted equilibrium fractions of the five polycyclic aromatic hydrocarbons in the three experimental conditions: bare (no serum), 2% fetal calf serum, bare (no serum) with Matrigel ECM hydrogel after 960min. The plot was generated from 50 bootstrap datasets by resampling the five experimental replicates with replacement. The model was refitted to each bootstrap dataset, and 95% percentile confidence intervals were calculated from the resulting equilibrium-fraction distributions. Bars show the best-fit predicted percentages of the nominal amount present as the free medium fraction, serum-bound fraction, plastic-bound fraction, or ECM-bound fraction, based on the loss-corrected data. Error bars represent 95% bootstrap confidence intervals.

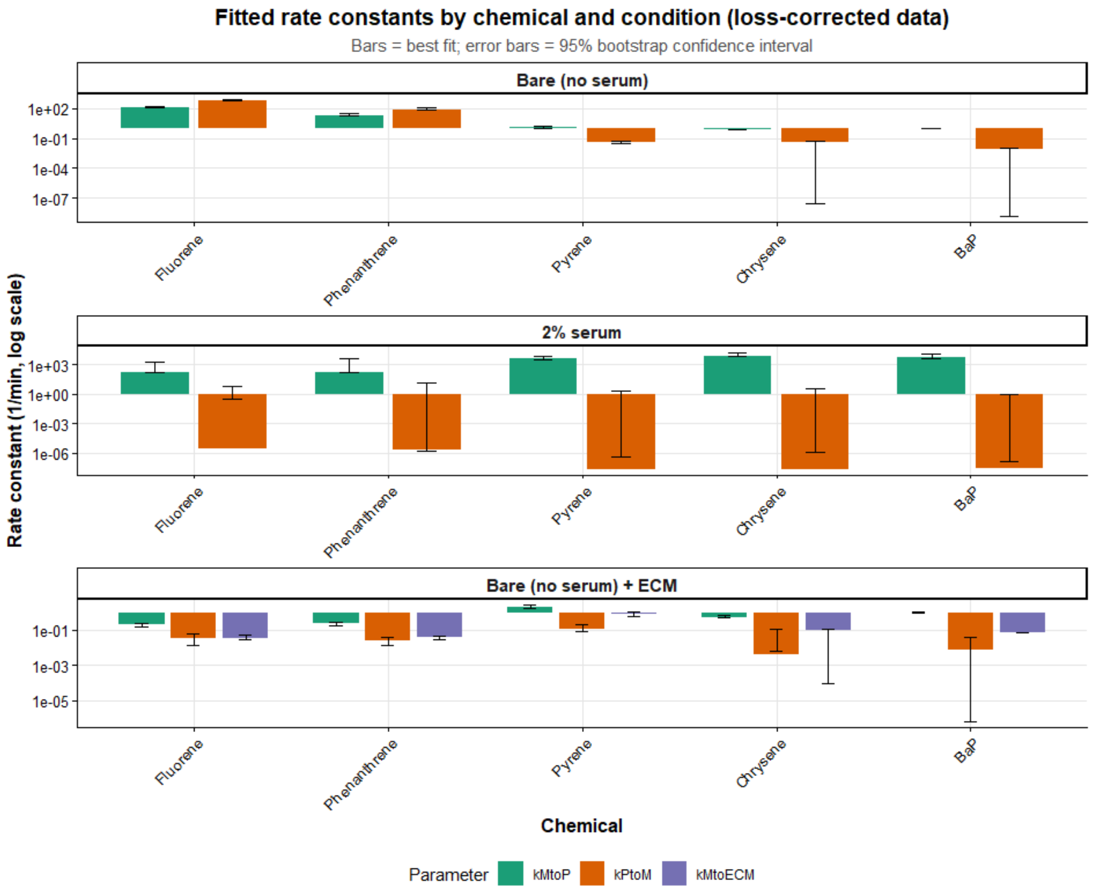

Supplementary Figure S5. Best-fit rate constants for the distribution of the five polycyclic aromatic hydrocarbons in the three experimental conditions: bare (no serum), 2% fetal calf serum, bare (no serum) with Matrigel ECM hydrogel after 960min. The plot was generated from 50 bootstrap datasets by resampling the five experimental replicates with replacement. The model was refitted to each bootstrap dataset, and 95% percentile confidence intervals were calculated from the resulting parameter and distributions. Bars show the estimated transfer-rate parameters obtained by fitting the compartmental model to the loss-corrected data: medium-to-plastic transfer ($k_{M\to P}$), plastic-to-medium transfer ($k_{P\to M}$),

and, where applicable, medium-to-ECM transfer ($k_{M \to ECM}$). Values are expressed in min^{-1} on a logarithmic scale. Error bars represent 95% percentile bootstrap confidence intervals based on 50 resampled datasets.
